# Tetanus following *Guboow* complications of Somali traditional burn therapy: a case report

**DOI:** 10.1186/s13256-025-05607-2

**Published:** 2025-10-21

**Authors:** Mulki Mukhtar Hassan, Mohamed Mustaf Ahmed, Najib Isse Dirie, Safia Nur Ali, Ruweida Abdi Mohamed

**Affiliations:** 1 Faculty of Medicine and Health Sciences, SIMAD University, Mogadishu, Somalia; 2 Department of Urology, Dr. Sumait Hospital, Faculty of Medicine and Health Sciences, SIMAD University, Mogadishu, Somalia

**Keywords:** Tetanus, *Guboow*, Traditional healing, *Clostridium tetani*, Somalia, Case report

## Abstract

**Background:**

Tetanus remains a significant public health concern, particularly in regions with low vaccination rates and prevalent traditional healing practices. *Guboow*, a Somali traditional healing practice involving intentional skin burns using heated objects, poses a high risk of infections, including tetanus, due to nonsterile conditions. This case highlights the complications arising from *Guboow* and emphasizes the importance of vaccination and culturally sensitive healthcare interventions.

**Case presentation:**

A 35-year-old Somali male, unvaccinated against tetanus, developed an infection 10 days after undergoing *Guboow*. The patient presented with respiratory distress, muscle stiffness, and trismus. Clinical examination revealed opisthotonus, burn ulcers, and autonomic instability. Laboratory investigations confirmed hypoxemia and mild hypernatremia. The patient was admitted to the intensive care unit and managed with mechanical ventilation, tetanus immunoglobulin, antibiotics, and wound care. Supportive intensive care unit management, including treatment for muscle spasms and autonomic dysfunction, led to a significant clinical improvement. By day 14 (post intensive care unit), the patient was successfully weaned off the ventilator, transferred to the general ward, and discharged without residual symptoms on day 24 (post intensive care unit).

**Conclusion:**

This case underscores the dangers of unsafe traditional healing practices and highlights the critical role of vaccination in tetanus prevention. There is an urgent need for culturally tailored public health education and collaboration with traditional healers to reduce the health risks associated with these unsafe practices. Further research is essential to assess the impact of traditional healing methods on infectious disease outcomes.

**Supplementary Information:**

The online version contains supplementary material available at 10.1186/s13256-025-05607-2.

## Introduction

Tetanus is a severe and life-threatening nervous system disease caused by the potent neurotoxin, tetanospasmin, produced by the anaerobic bacterium *Clostridium tetani* [1]. This toxin inhibits the release of inhibitory neurotransmitters, such as gamma-aminobutyric acid (GABA) and glycine, within the spinal cord and brainstem, leading to characteristic painful muscle rigidity and spasms [2]. *C. tetani* spores are widely present in soil, dust, and animal feces, typically entering the body through wounds—even minor abrasions, punctures, or burns [2]. The incubation period for tetanus varies, commonly ranging from 3 to 21 days, but can extend from 1 day to several months; generally, a shorter incubation period is associated with more severe disease [3]. Despite being an entirely vaccine-preventable disease, tetanus remains a significant public health challenge, particularly in developing countries [2]. The World Health Organization (WHO) reported 12,476 global cases in 2017 [4], and a systematic review and meta-analysis found the pooled crude case fatality rate for adult tetanus in African care facilities to be 43.2% [5].

This report presents a rare yet critical instance of generalized tetanus in an adult, uniquely linked to an unsafe traditional healing practice known as *Guboow* [6]. In many parts of Somalia, *Guboow* (meaning “scar” or “burning” in Somali) is a traditional practice involving the deliberate burning of body parts, often for therapeutic purposes [6, 7]. This practice carries significant health risks, particularly when invasive or performed in unsanitary conditions, as it can lead to severe infections and other complications [6]. Such practices can serve as direct portals of entry for *C. tetani* spores, leading to life-threatening infections like tetanus [8, 9]. This case further highlights the persistent challenge of low adult tetanus immunization coverage in regions like Somalia, where conflict, poverty, and limited healthcare access contribute to inadequate vaccination rates [10]. The disproportionate affliction of men by tetanus in these areas is often linked to their occupational exposures and lower immunization rates compared with women [11]. This report aims to detail a rare case of generalized tetanus in an adult acquired through the traditional Somali healing practice of *Guboow*, highlighting its clinical presentation, management challenges, and public health implications in a low-resource setting. This case report has been prepared in accordance with the Case Report (CARE) guidelines to ensure comprehensive and transparent reporting. The CARE checklist has been included as a supplementary file (Supplementary Material S1).

## Case presentation

We report a case of a 35-year-old unvaccinated Somali male with no history of chronic diseases who was referred to the intensive care unit (ICU) from another hospital owing to respiratory distress and generalized body stiffness that had persisted for 5 days prior to hospitalization. His medical history revealed that 10 days before admission, he had undergone *Guboow* (traditional burn therapy) performed by a healer. The patient was a construction worker, and community members believed that his neck swelling indicated mumps, a condition commonly treated in Somalia with *Guboow*, a traditional cauterization practice rooted in local beliefs. The patient did not seek medical care or receive tetanus vaccination at that time. By day 5 post burn, he developed muscle stiffness and trismus, making it difficult for him to open his mouth.

He presented to another hospital, where neck ultrasound suggested sialadenitis, which was conservatively managed. This initial diagnosis may have contributed to a delay in recognizing the early signs of tetanus, such as trismus and muscle stiffness, highlighting the diagnostic challenge in differentiating infectious from neurological causes of neck swelling and rigidity in the early stages. However, over the next few days, his symptoms worsened, with muscle rigidity developing in the neck, back, and abdomen, progressing to generalized stiffness. Owing to the progressive severity of his symptoms, he was transferred to the ICU for further management. On day 10 post burn (hospital admission day), the patient was diaphoretic and had difficulty speaking owing to jaw stiffness. He exhibited respiratory distress with vital signs notable for hypertension (blood pressure [BP] 143/92 mmHg), tachycardia (125 beats/minute), tachypnea (36 breaths/minute), and hypoxia (SpO_2_ 85% on a non-rebreather mask). He was afebrile. Neurologically, the patient exhibited trismus and risus sardonicus.

**Fig. 1:**
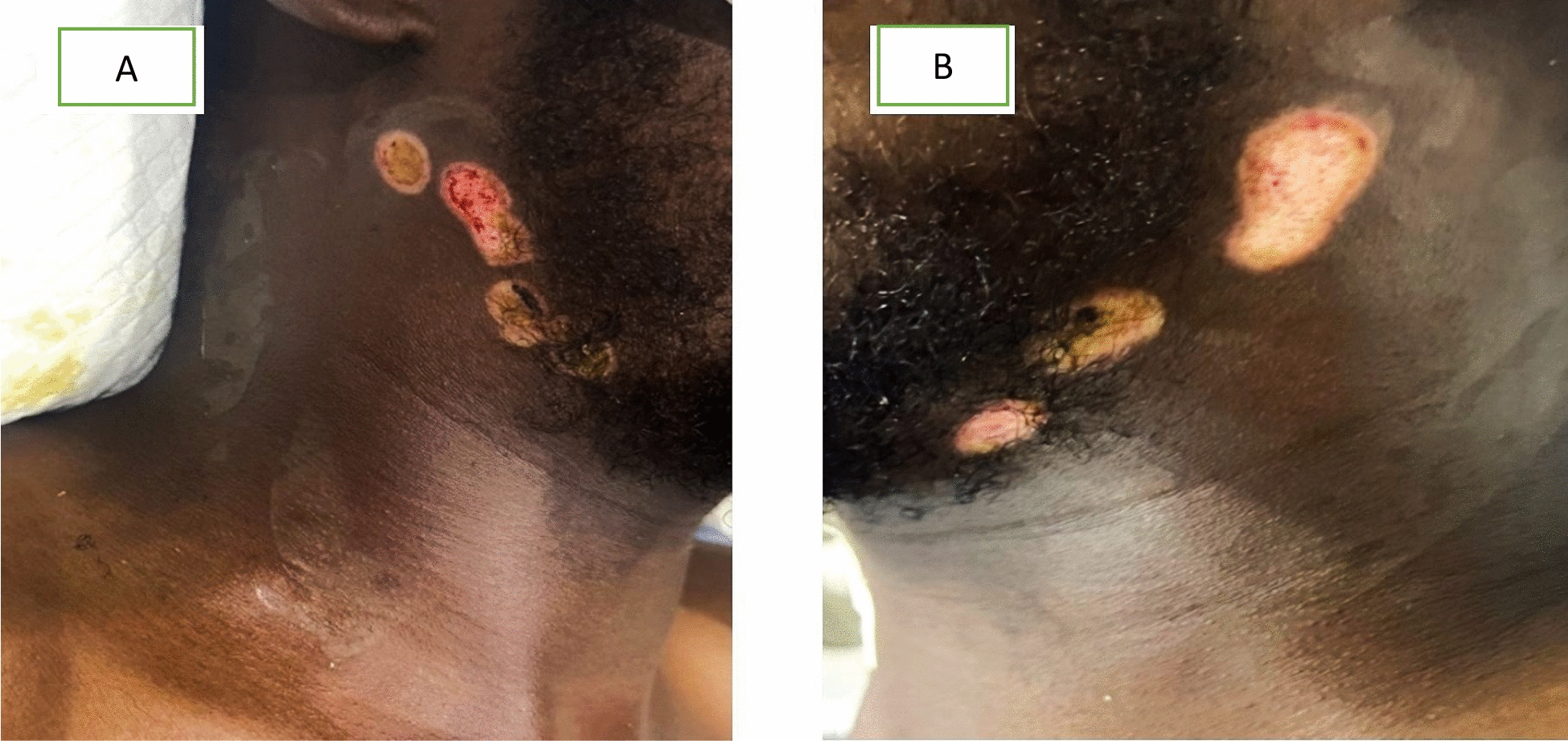
Neck lesions on the right (**A**) and left (**B**) sides resulting from *Guboow*, a traditional Somali burn therapy. The images show multiple circular second-degree burns measuring approximately [insert size, for example, 3–5 cm in diameter] and characterized by necrotic and erythematous tissue with areas of eschar formation. There were no overt signs of purulent discharge, but the lesions served as entry points for *Clostridium tetani*, leading to generalized tetanus infection

In addition, there were multiple circular second-degree burns with necrotic and erythematous areas on both sides of the neck (Fig. 1). Initial laboratory tests were unremarkable except for arterial blood gas analysis, which showed hypoxemia, and serum electrolytes, which revealed mild hypernatremia. During hospitalization, the patient’s hemoglobin level decreased from 11.0 to 9.9 g/dL. This mild anemia could be multifactorial, potentially resulting from hemodilution, frequent blood sampling, or an underlying inflammatory response associated with infection and critical illness (Table 1). On the basis of the clinical presentation and history, a diagnosis of generalized tetanus was made, and the patient was immediately intubated and placed on mechanical ventilation owing to worsening respiratory distress. On day 10 (ICU admission day), the patient was intubated and started on mechanical ventilation. He was treated with midazolam (5 mg/hour), rocuronium (10–15 mg/hour), and fentanyl (0.25 mcg/kg/hour) to control severe spasms and pain.

**Table 1:** Temporal changes in key laboratory parameters from admission to day 14

Laboratory parameter	Reference range	Value on admission	Value at day 14
pH	7.35–7.45	7.361	7.46
PCO_2_ (mmHg)	35–45	29.6	34.8
PO_2_ (mmHg)	80–100	50	85
HCO_3_^−^ (mmol/L)	22–28	16.3	24
WBC (× 10^9^/L)	4.0–11.0	12.72	11.98
Hemoglobin (g/dL)	13.5–17.5 (male)	11.0	9.9
Platelets (× 10^9^/L)	150–400	198	396
Sodium (mmol/L)	135–145	148.9	138.7
Potassium (mmol/L)	3.5–5.0	3.46	5.09
Calcium (mmol/L)	2.2–2.6	2.32	2.35
Creatinine (mg/dL)	0.6–1.3	0.95	0.88
Blood urea (mg/dL)	7–20	49.54	26.08
ALT (U/L)	7–56	22.2	19.6
AST (U/L)	10–40	41.6	32.9
CK (U/L)	26–192	Not done	1354

*PCO*_2_ partial pressure of carbon dioxide,*PO*_2_ partial pressure of oxygen, *HCO*_3_ bicarbonate, *WBC* white blood cell count, *HGB* hemoglobin, *PLT* platelet count, *ALT* alanine aminotransferase, *AST* aspartate aminotransferase, *CK* creatine kinase

Although the patient received 3000 IU of human tetanus immunoglobulin (HTIG), current guidelines recommend a lower dose of 500 IU, which has been demonstrated to be equally effective in neutralizing tetanospasmin while reducing adverse events and costs. In this case, a higher dose was administered on the basis of the hospital’s existing protocol at the time of treatment. He also received intramuscular human tetanus immunoglobulin (HTIG; 3000 units) and intravenous metronidazole (500 mg every 8 hours) treatment. Wound debridement and dressing were performed to prevent secondary infections.

Magnesium sulfate (MgSo_4_) therapy was initiated to control muscle spasms and autonomic dysfunction, aiming to reduce the need for muscle relaxants and sedatives. The patient received a 4-g intravenous loading dose of MgSO_4_, followed by a maintenance infusion of 2–3 g/hour, adjusted according to the clinical response and monitored magnesium and calcium levels. His condition gradually improved over the following days. By ICU day 10, significant clinical improvement allowed for the gradual tapering of midazolam and discontinuation of rocuronium.

By ICU day 14, the patient was breathing spontaneously and had regained adequate consciousness. On ICU day 15, sustained clinical improvement enabled successful extubation. He was transferred from the ICU to the general ward shortly thereafter and was discharged in a stable condition without residual neurological deficits. Prior to discharge, the patient was educated about wound care, hygiene, and the dangers of traditional practices. He was administered a tetanus toxoid vaccine and was referred for follow-up in the outpatient clinic to ensure completion of the full vaccination series. A detailed timeline of clinical events and interventions from ICU admission to discharge is presented in Table 2.

**Table 2:** Timeline of clinical events and interventions during hospitalization

Hospital day	Clinical event/intervention
Day 0	ICU admission; patient intubated and started on sedation, mechanical ventilation, and empirical antibiotics. Received 3000 IU intramuscular human tetanus immunoglobulin (HTIG)
Day 1–3	Continued intravenous metronidazole therapy. Spasms persisted despite supportive care
Day 4	Magnesium sulfate initiated for spasm control; partial improvement observed
Day 5–9	Gradual reduction in muscle spasms noted
Day 10–14	Ongoing supportive ICU care; attempts made to taper magnesium sulfate and assess readiness for ventilator weaning. Laboratory tests on day 14 indicated clinical improvement
Day 15	Patient successfully extubated; respiratory status remained stable
Day 16–20	Continued observation in ICU post extubation; patient stable and tolerating oral feeding
Day 21	Transferred to step-down/ward-level care (private room)
Day 24	Discharged home in stable condition. Received tetanus vaccination prior to discharge and received counseling on wound care and traditional practice risks

## Discussion

This case highlights a rare yet critical instance of generalized tetanus in an adult, directly linked to an unsafe traditional healing practice known as *Guboow*. Tetanus, a severe nervous system disease caused by *Clostridium tetani*, presents with painful muscle rigidity and spasms, and despite being vaccine-preventable, remains fatal in many developing countries [1]. The patient’s symptoms were classic for generalized tetanus, including neck and jaw stiffness and difficulty swallowing [9]. A key success in this case was the effective use of magnesium sulfate (MgSO_4_), which is commonly employed to manage autonomic dysfunction and was noted to significantly reduce the need for sedatives and muscle relaxants in the intensive care unit (ICU) [2]. Despite a delay in seeking orthodox treatment after the injury, the comprehensive ICU management provided ultimately led to a positive outcome for the patient [12].

This case critically highlights the persistent challenge of low adult tetanus immunization coverage in regions like Somalia [10], which contributes to tetanus remaining a significant public health burden in Africa [5]. A substantial proportion of patients with tetanus in Africa have no history of vaccination [11]. The patient’s failure to receive post-exposure prophylaxis or a tetanus shot after his tetanus-prone injury was a missed opportunity for vaccination [12]. While childhood immunization coverage has increased globally, adult and adolescent men, in particular, have been largely missed by vaccination programs [11]. Healthcare providers must be educated on the substantial health risks associated with high-risk traditional practices that break the skin, such as *Guboow*, which can serve as a direct portal of entry for *C. tetani* spores and lead to severe infections [8, 9].

Traditional healing practices are deeply embedded in the cultural context of many communities in Somalia and other parts of Africa, often serving as the primary source of healthcare where conventional medicine is limited [6]. Practices like *Guboow* (which involves deliberate burning) hold cultural significance [7]. They unfortunately carry significant health risks, especially when invasive or unsanitary [7]. Such practices can result in severe local infections, extensive skin defects, and even life-threatening conditions like sepsis [6, 7]. It is crucial for healthcare professionals to approach this issue with cultural sensitivity, while engaging patients and caregivers in respectful education about the risks [7], as *Guboow* itself has been described as a “harmful practice or even abuse” [6].

On the basis of the insights from this case and the broader literature, several recommendations have emerged to reduce the burden of tetanus. First, all adult patients with tetanus-prone injuries should receive the tetanus and diphtheria (Td) vaccine [2], even if human tetanus immune globulin (HTIG) is administered [2], as tetanus infection does not confer natural immunity and booster doses are essential for lifelong protection [3]. Second, emergency protocols in low- and middle-income countries (LMICs) should emphasize early ICU referral for suspected tetanus cases, recognizing the high mortality in resource-limited settings where mechanical ventilation is often unavailable or unaffordable [5]. Countries like Senegal, through their Expanded Vaccination Programs (EVP), have achieved significant progress in eliminating maternal and neonatal tetanus, offering a model for Somalia to strengthen its national immunization programs by enhancing adherence to EVP, with particular emphasis on booster vaccinations [4]. Concurrently, comprehensive education on traditional wound care complications is vital for both healthcare providers and the general public [9]. This includes provider training in recognizing complications from traditional practices [8, 9]. Finally, healthcare systems should prioritize integrating immunization into hospital discharge planning to ensure high-risk individuals receive necessary vaccinations and actively work to improve vaccine accessibility through culturally appropriate outreach programs [10].

## Conclusion

This case highlights a preventable, life-threatening infection stemming from unsafe traditional healing practices such as *Guboow*. The patient experienced successful intensive care management with magnesium sulfate, which led to a favorable outcome. A critical learning point is the missed opportunity for tetanus vaccination at the time of the injury. This case underscores the urgent need for clinician and community education on the risks of tetanus and the importance of safe wound-care practices. Healthcare systems in Somalia should strengthen vaccine accessibility and promote culturally sensitive public health education.

## Supplementary Information

Supplementary material 1.

## Data Availability

The data supporting the findings of this study are included in the manuscript.
