# Supplementary material for: Tetanus following Guboow complications of Somali traditional burn therapy: a case report
Source: J Med Case Rep. 2025 Oct 21;19:529. doi: 10.1186/s13256-025-05607-2 (PMC12542510; doi:10.1186/s13256-025-05607-2)
Supplement: Supplementary file 1 — Supplementary material 1. [file 13256_2025_5607_MOESM1_ESM.pdf]

CARE case report guidelines

CARE Checklist of information to include when writing a case report

(cc) BY-NC-ND

Topic	Item	Checklist item description	Reported on Line
Title	1	The diagnosis or intervention of primary focus followed by the words "case report"	Line 1- 2
Key Words	2	2 to 5 key words that identify diagnoses or interventions in this case report, including "case report"	Line 53
Abstract (no references)	3a	Introduction: What is unique about this case and what does it add to the scientific literature?	Line 78 - 80
	3b	Main symptoms and/or important clinical findings	Line 37 - 39
	3c	The main diagnoses, therapeutic interventions, and outcomes	Line 39 - 43
	3d	Conclusion—What is the main "take-away" lesson(s) from this case?	Line 230 - 231
Introduction	4	One or two paragraphs summarizing why this case is unique (**may include references**)	Line 78 - 85
Patient Information	5a	De-identified patient specific information.	Line 89
	5b	Primary concerns and symptoms of the patient.	Line 89 - 98
	5c	Medical, family, and psycho-social history including relevant genetic information	Line 89 - 95
	5d	Relevant past interventions with outcomes	Line 100 - 104
Clinical Findings	6	Describe significant physical examination (PE) and important clinical findings.	Line 107 - 111
Timeline	7	Historical and current information from this episode of care organized as a timeline	Line 89 - 157
Diagnostic Assessment	8a	Diagnostic testing (such as PE, laboratory testing, imaging, surveys).	Line 89 - 157
	8b	Diagnostic challenges (such as access to testing, financial, or cultural)	Line 100 - 104
	8c	Diagnosis (including other diagnoses considered)	Line 120
	8d	Prognosis (such as staging in oncology) where applicable	
Therapeutic Intervention	9a	Types of therapeutic intervention (such as pharmacologic, surgical, preventive, self-care)	Line 123 - 147
	9b	Administration of therapeutic intervention (such as dosage, strength, duration)	Line 123 - 147
	9c	Changes in therapeutic intervention (with rationale)	Line 143 - 147
Follow-up and Outcomes	10a	Clinician and patient-assessed outcomes (if available)	
	10b	Important follow-up diagnostic and other test results	
	10c	Intervention adherence and tolerability (How was this assessed?)	
	10d	Adverse and unanticipated events	
Discussion	11a	A scientific discussion of the strengths AND limitations associated with this case report	Line 180 - 185
	11b	Discussion of the relevant medical literature **with references**.	Line 175 - 206
	11c	The scientific rationale for any conclusions (including assessment of possible causes)	Line 175 - 197
	11d	The primary "take-away" lessons of this case report (without references) in a one paragraph conclusion	Line 228 - 235
Patient Perspective	12	The patient should share their perspective in one to two paragraphs on the treatment(s) they received	
Informed Consent	13	Did the patient give informed consent? Please provide if requested	Yes ☑ No ☐
